# Does the Mode of Anaesthesia (General or Regional) Affect Survival and Complications Following Femoropopliteal and Femorodistal Bypass?

**DOI:** 10.7759/cureus.32104

**Published:** 2022-12-01

**Authors:** Malin Gunawardena, Mohammad Salami, Adam Howard, Ayoola Awopetu

**Affiliations:** 1 Vascular Surgery, East Suffolk and North Essex NHS Foundation Trust, Colchester, GBR

**Keywords:** general anaesthesia, regional anaesthesia, peripheral vascular disease, spinal anaesthesia, emergency and elective surgery, peripheral angioplasty and stenting, bypass graft, general and vascular surgery, pvd: peripheral vascular disease, peripheral vascular surgery

## Abstract

Introduction: Femoropopliteal and femorodistal bypasses are indicated in the management of severe claudication or critical limb ischaemia. The choice of type of anaesthesia between general anaesthesia (GA) and regional anaesthesia (RA; epidural/spinal) has remained controversial. The study aimed to compare the rates of graft failure, perioperative mortality, and other major complications (including graft failure) associated with GA versus RA for lower limb bypass revascularisation in patients presenting with significant peripheral arterial disease.

Methods: All patients who had femoropopliteal and femorodistal bypass at the vascular unit of Colchester Hospital between January 2016 and September 2018 were included. The primary outcome measure was survival, and secondary outcome measures were respiratory, wound, cardiac, and graft failure complications. Technical success was defined as a successful operation with demonstrated graft patency at discharge and up to 30 days of follow-up. Secondary outcome measures included respiratory, wound, and cardiac complications. Statistical analysis included descriptive statistics and tests of association using chi-square for graft failure outcomes and the Mann-Whitney U test for the length of stay. All analyses were done at a 5% level of significance.

Results: There were 139 patients identified during the study period who fulfilled the inclusion criteria, of which 127 had complete records. The overall mortality and morbidity in this study were 1.6% and 14.229%, respectively. The proportion of patients who had ischaemic heart disease is almost threefold amongst those who had failed bypass compared to the successful bypass group (33.3% versus 11.9%, p = 0.018). A total of 65 patients received GA and 62 patients underwent RA; there were no significant differences in baseline preoperative comorbidities, postoperative mortality and complications, and length of stay. The majority (84%) of the patients who had RA had combined spinal and epidural (CSE) anaesthesia. The overall mortality and morbidity in this study were 1.6% and 14.2%, respectively. The proportion of patients with graft failure was 14.5% for GA versus 13.8% for RA (p = 0.914); there was no significant difference for conduit type, quality of vein conduit, the prevalence of heart failure and postoperative hypotensive episodes, redo-surgery, and major amputation, and length of stay (GA: 5.0, RA: 6.0, p = 0.854) did not differ significantly between the two groups. The proportion of patients who had ischaemic heart disease is almost threefold amongst those who had failed bypass compared to the successful bypass group (33.3% versus 11.9%, p = 0.018).

Conclusion: The mode of anaesthesia, GA or the use of CSE RA in approximately half of the patients, did not influence survival, respiratory, cardiac, wound, graft failure, or length of stay in this study. There was a relationship between the presence of cardiac comorbidity and bypass failure, indicating a need for a standard care protocol to further optimise cardiac perioperative care to improve outcomes.

## Introduction

Infrainguinal bypass procedures usually done under general anaesthesia (GA) or regional anaesthesia (RA) (epidural/spinal) are indicated in the management of peripheral arterial disease (PAD) with disabling functional ischaemia or in the setting of critical limb ischaemia (CLI) defined as a threatened limb with rest pain or tissue loss [1]. PAD is a manifestation of atherosclerosis characterised by stenosis and/or occlusion of large and medium-sized arteries, estimated to affect more than 200 million people globally, including one in five people over the age of 60 years in the United Kingdom (UK) [2]. PAD carries the risk of lower limb loss and is also a risk factor for adverse cardiovascular events, including an increased risk of death from heart attack and stroke [2,3]. Femoropopliteal and femorodistal bypasses using an autogenous vein or prosthetic grafts reroute blood flow to poorly perfused distal sites, circumventing vessel occlusions. The spectrum of patients amenable to surgical revascularisation includes patients stratified by the Trans-Atlantic Inter-Society Consensus (TASC) for the Management of Peripheral Arterial Disease into categories C and D based on the anatomic complexity and location, and clinical status of the patient [4].

The decision to pursue surgical, endovascular, or combined approaches to revascularisation is tailored to the patient (anaesthetic risk, severity of comorbid conditions), the pattern of occlusive disease, the extent of tissue loss, previous failed interventions, and the goals of care. In patients with multi-level femoropopliteal disease, defined as TASC C or D, and who have a good-quality venous conduit, surgical revascularisation remains the management modality of choice with increasing contention for angioplasty as a first argument [5]. This scenario often leads to more complex bypass procedures following failed attempts at endovascular treatment and calls for efforts to seek factors that can improve outcomes. One of these approaches includes assessing the influence of anaesthesia on the outcome of vascular bypass.

The combined spinal-epidural (CSE) technique is used to add the advantage of the reliability and early onset of spinal anaesthesia to the additional benefit of continuous epidural block [6]. Stress response attenuation is reported to be an advantage of spinal anaesthesia due to the avoidance of hyperdynamic responses to tracheal intubation and extubation and lower levels of stimulated catecholamines [1,6]. Studies have also shown increased thromboelastographic markers and plasminogen activator inhibitor-1 (PAI-1) levels in patients receiving GA compared to epidural anaesthesia or CSE anaesthesia [7,8]. These anti-thrombotic influences suggest a clinical advantage for RA in agreement with the findings of a lower rate of graft occlusion among patients receiving epidural anaesthesia in a prospective randomized trial of 100 patients by Christopherson et al. [9]. The clear benefit of the decreased risk of regrafting or thrombectomy in that study (4% versus 22% for epidural) led to the decision to terminate the study early [9]. However, it should be noted that premature termination of clinical trials has been reported to be a form of bias shown to be prone to overestimation of treatment effect [10]. Dodds et al. also reported a significant reduction in early graft failure among patients randomised to epidural anaesthesia, and the difference in graft failure was not sustained at 30 days [11].

Several studies, however, have shown no difference in graft function, limb salvage rate, and cardiac morbidity or mortality between RA and GA [12-16]. Furthermore, the recently updated Cochrane review, which included four clinical trials with a total number of 696 participants, showed no difference between participants who had neuraxial anaesthesia or GA with respect to mortality rate, myocardial infarction, and lower-limb amputation [17]. Nonetheless, the review identified a significant reduction in the incidence of pneumonia after neuraxial anaesthesia. The authors, therefore, concluded that enough evidence is not available to rule out clinically significant differences between the two modes of anaesthesia for patients undergoing infrainguinal bypass, leaving room for further studies. This study was therefore aimed at comparing the rates of graft failure, perioperative mortality, and other major complications associated with GA versus RA for lower limb bypass revascularisation in patients presenting with PAD.

## Materials and methods

A retrospective, observational, descriptive, comparative study was performed as part of an audit of procedures done at the Five Rivers Vascular Unit of the Colchester Hospital for femoropopliteal and femorodistal bypass. The clinical records of all patients who had femoropopliteal and femorodistal bypass for peripheral arterial disease between January 2016 and September 2018 were collected from the hospital database. These included patients presenting with disabling claudication, critical limb ischaemia, and acute limb ischaemia. The patients were operated on as emergency, urgent, or elective. Patients who had acute popliteal artery aneurysms or acute re-do operations on the same limb were excluded from this study. The audit department at Colchester Hospital approved the use of the anonymised data for this study.

Patient characteristics and outcome measures

Patients’ baseline demographic characteristics and comorbidities were collected, including age, sex, race, the presence of hypertension, diabetes, coronary artery disease, congestive heart failure (CHF), chronic obstructive pulmonary disease (COPD), end-stage renal disease (ESRD), and American Society of Anaesthesiologists (ASA) classification. In addition, other potential contributing factors, such as the use of antiplatelet regimens (aspirin and clopidogrel) and operative characteristics (case urgency, type of conduit, origin and destination of the bypass), were also included. The mode of anaesthesia pre- and post-bypass was recorded. Procedural details and 30-day outcomes were analysed. Technical success was defined as a successful operation with demonstrated graft patency at discharge and up to 30 days follow-up measured using Doppler or duplex studies. The primary outcome measure was procedural success (30-day graft patency). Secondary outcome measures included respiratory compromise, wound and cardiac complications, perioperative mortality, estimated blood loss, length of stay, new-onset myocardial infarction (MI), new-onset CHF, and acute kidney injury (AKI). The complications were graded using the classification method by Dindo et al., with significant morbidity being grade II-IV [18].

Anaesthesia and surgical methods

Anaesthesia

Spinal anaesthetic consisted of 0.5% heavy Marcaine with or without fentanyl. For the CSE, the spinal is first given as described, then 90-120 minutes later, the epidural catheter is topped up with 0.25-0.375% Chirocaine, titrated to maintain a dermatome level between T8-10. GA was induced with propofol and fentanyl (1-5 µg/kg IV), and the muscle relaxant was either atracurium or rocuronium. After intubation, anaesthesia was maintained with oxygen, air, sevoflurane or desflurane. Boluses of fentanyl or morphine were given as required. Vasoactive drugs and fluids were administered at the discretion of the anaesthesia care team managing the patient.

Surgery

After induction of anaesthesia, the long saphenous or arm vein was exposed by a single continuous incision for the adequate length required. The femoral and outflow arteries were then exposed. Saphenous vein grafts were generally tunnelled anatomically to the popliteal artery, crural, or pedal level. The veins were either used reversed or had their valves cut (with a valvulotome) and used non-reversed if this configuration better matched the diameter differences between the inflow and outflow arteries.

Prosthetic grafts were polytetrafluoroethylene (PTFE) or Distaflo® grafts depending on surgeon preference and the surgical anatomy. All anastomoses were performed with polypropylene (5-0, 6-0, or 7-0) suture under loupe magnification. Heparin (80-100 U/kg) was administered to all patients during the operation and was not reversed at the end of the procedure. Postoperative antiplatelet and anticoagulation consisted of aspirin or clopidogrel (75 mg orally per day) and enoxaparin while bedridden for vein grafts.

Statistical analysis

Data were analysed based on the type of anaesthetic received. Baseline patient demographics, perioperative risk factors, surgical indications, type of conduit, outflow target artery, the incidence of postoperative morbidity, bypass failure, and other outcomes were compared between the RA and GA groups. Statistical analysis using SPSS (version 24, IBM Corp., Armonk, NY) included descriptive statistics using frequency tables, percentages, means, standard deviation (SD), median, and interquartile range (IQR), and tests of association using chi-square for graft failure outcomes and Mann-Whitney U test for the length of stay. All analyses were done at a 5% level of significance.

## Results

Patient and operative characteristics

**Figure 1:**
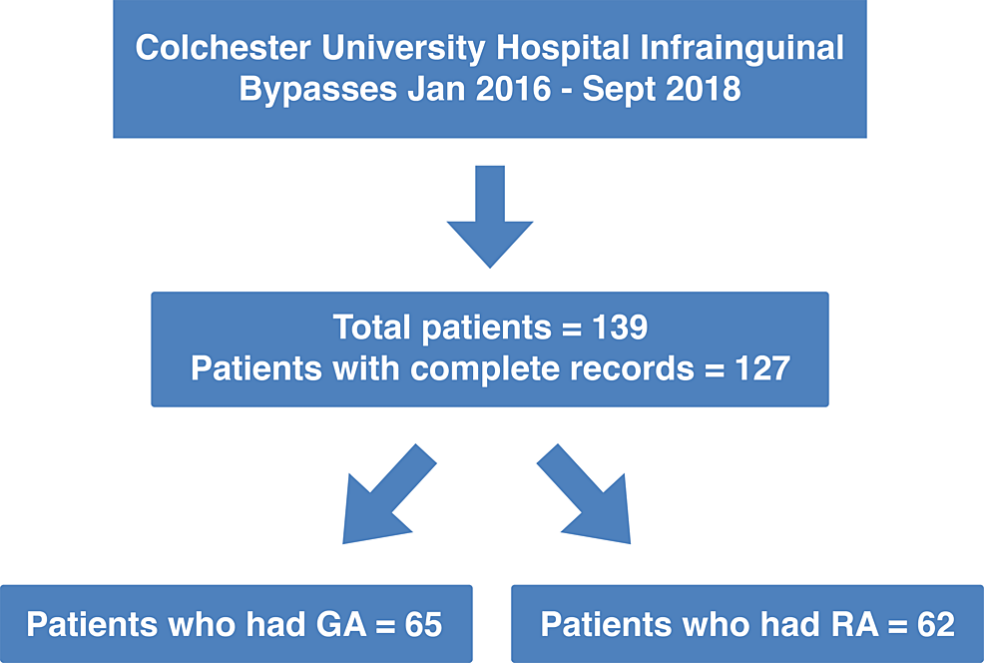
Distribution of patients identified to have general anaesthesia (GA) or regional anaesthesia (RA) for infrainguinal bypass

There were 139 patients identified during the study period who fulfilled the inclusion criteria. However, complete data were available for only 127 patients with 14 of the total cohort missing full surgical and anaesthetic records (Figure 1). The baseline characteristics of the patients in comparison groups are presented in Table 1. The study groups showed similar preoperative characteristics including age, presence of diabetes, hypertension, and chronic obstructive pulmonary disease. A total of 17 (13.4%) patients had recent or failed attempts at angioplasty. Out of the 22 patients who had failed bypasses, four patients also had missing detailed operative records. The majority (52, 84%) of the patients who had RA had CSE anaesthesia.

**Table 1:** Baseline characteristics of patients undergoing lower extremity bypass under general anaesthesia (GA) and regional anaesthesia (RA)

	GA (n = 65)	RA (n = 62)
Male	49 (38.6%)	41 (32.3%)
Female	16 (12.6%)	21 (16.5%)
Age, in years (mean ± SD)	73.49 ± 13.3	74.32 ± 13.3
Age ≥ 80 years	24 (18.9%)	22 (17.3%)
Age < 80 years	41 (32.3%)	40 (31.5%)
Hypertension	32 (25.2%)	39 (30.7%)
BMI, in kg/m2 (mean ± SD)	27.24 ± 4.8	26.95 ± 5.04
Diabetes	19 (15.0%)	20 (15.7%)
Ischaemic heart disease	7 (5.5%)	9 (7.1%)
Hypercholesterolemia	5 (3.9%)	8 (6.3%)
Chronic obstructive pulmonary disease	15 (11.8%)	15 (11.8%)
End-stage renal disease	7 (5.5%)	8 (6.3%)
Recent/failed angioplasty	11 (8.7%)	6 (4.7%)

Operative characteristics were also analysed for any significant differences (Table 2). The most common indications for bypass were rest pain (37.7%) and tissue loss (35.3%) (gangrene and ulcers) in both arms of this study. Overall, there was no significant difference in terms of the indications for surgery between the two groups. Bypass graft origin was from the common femoral artery in most cases (95.3%) whereas graft destination had greater variability, with more than half (63%) of the population in both groups having a popliteal landing zone. A total of 72 patients (56.7%) had elective bypass procedures.

**Table 2:** Operative characteristics of patients undergoing lower limb bypass surgery with general anaesthesia (GA) and regional anaesthesia (RA)

		GA (n = 65)	RA (n = 62)
Indication	Disabling claudication	16 (13.1%)	17 (13.9%)
	Rest pain	20 (16.4%)	26 (21.3%)
	Gangrene	9 (7.4%)	6 (4.9%)
	Ulcer (tissue loss)	18 (14.8%)	10 (8.2%)
Graft origin	Femoral artery	62 (48.8%)	59 (46.5%)
	Profunda artery	1 (0.8%)	3 (2.4%)
	Popliteal artery	2 (1.6%)	0 (0.0%)
Graft recipient	Popliteal	43 (33.9%)	37 (29.1%)
	Tibioperoneal trunk	2 (1.6%)	4 (3.1%)
	Anterior tibial	9 (7.1%)	6 (4.7%)
	Posterior tibial	5 (3.9%)	8 (6.3%)
	Peroneal	6 (4.7%)	7 (5.5%)
Urgency of case	Elective	38 (29.9%)	34 (26.8%)
	Urgent	25 (19.7%)	25 (19.7%)
	Emergent	2 (1.6%)	3 (2.4%)
Conduit type	Autogenous	57 (44.9%)	58 (45.7%)
	Prosthetic	8 (1.2%)	4 (0.6%)

Complications included graft failure or thrombosis, excessive haemorrhage necessitating blood transfusion, wound infection and dehiscence, wound seroma/haematoma, and two incidences of postoperative MI resulting in death (Table 3).

**Table 3:** Distribution of postoperative complications

Complication	Frequency
Graft failure or thrombosis	18
Major haemorrhage with post-anaemia and transfusion	7
Wound infection and dehiscence	8
Pneumonia	2
Sepsis	1
Amputations above the ankle	9
Postoperative wound haematoma/seroma	4
Reoperation for revision of bypass or thrombectomy	4
Compartment syndrome	1
Graft stenosis	1
Myocardial infarction	2
Mortality	2
Severe postoperative hypotension	2

Graft Failure

There were a total of 18 (14.2%) graft failures and nine of these occurred in the group of patients that had GA. The type of anaesthesia did not affect graft failure at 30 days (p > 0.05; Table 4).

**Table 4:** Outcome at 30 days by type of anaesthesia received GA: general anaesthesia; RA regional anaesthesia; IQR: interquartile range.

Outcome	GA (n = 65)	RA (n = 62)	P-value
Graft failure	9 (14.5%)	9 (13.8%)	0.914
Perioperative mortality	1 (1.5%)	1 (1.6%)	
Length of stay (days) median (IQR)	5.00 (5)	6.00 (6)	0.854
Significant morbidity	19 (29.2%)	18 (29.0%)	0.9809
Revised grafting or thrombectomy	2 (3.1%)	2 (3.2%)	0.962
Amputation above the ankle	3 (9.2%)	6 (4.8%)	0.335

Related Return to the Operating Room

There were a total of 13 (10.2%) patients who had to be returned to theatre for re-do operations, i.e., revised grafting, thrombectomy, or amputations.

Surgical Length of Stay

The average length of stay (LOS) for patients undergoing GA was five days (interquartile range (IQR) = 5) and for RA, it was six days (IQR = 6). Mann-Whitney U test showed no significant difference in the postoperative LOS between GA and RA.

Again, the anaesthesia mode did not appear to have any influence on bypass failure across different locations for bypass types (Table 5).

**Table 5:** Outcome for bypass types stratified by mode of anaesthesia

	Bypass type	Successful	Failed	P-value
Regional	Femoropopliteal	27 (84.4%)	5 (15.6%)	0.515
	Femorodistal	21 (84.0%)	4 (16.0%)	
	Popliteal pedal	5 (100.0%)	0 (0.0%)	
	General	Bypass type	Successful	Failed	
	Femoropopliteal	36 (87.8%)	6 (12.2%)	0.637
	Femorodistal	17 (85.7%)	3 (14.3%)	
	Popliteal pedal	3 (100.0%)	0 (0.0%)	

Failed bypass

The distribution of comorbidities, operative factors, and immediate outcomes for patients with failed bypass as compared to those who had successful bypass is shown in Table 6. The proportion of patients with hypertension amongst patients who had a failed bypass was similar compared to the successful bypass group at about 50% and 57.9%, respectively. A higher percentage of patients who had failed bypass had diabetes (44.4% versus 28.0%) but this did not reach statistical significance. The proportion of patients who had ischaemic heart disease was almost threefold compared to the successful bypass group (33.3% versus 11.9%, p = 0.018), which was statistically significant. Critical limb ischaemia was the predominant presentation pattern both in the failed bypass group and the successful bypass group, though with a slightly increased proportion of patients with CLI in the failed bypass group (77.8% versus 74.9%). Within the group of patients with failed bypass, there was no significant statistical difference in the prevalence of heart failure, atrial fibrillation, quality of vein conduit, and hypotensive episodes in the first postoperative 48 hours.

**Table 6:** Distribution of failed bypass and comparison with successful bypass group ATA = anterior tibial artery; PTA = posterior tibial artery; BKA = below knee amputation; AKA = above knee amputation.

		Failed (n = 18)	Successful (n = 109)	P-value
Comorbidities	Hypertension	9 (50%)	62 (57.9%)	0.189
	Diabetes	8 (44.4%)	30 (28.0%)	0.088
	Ischaemic heart disease	6 (33.3%)	13 (11.9%)	0.018
	End-stage renal disease	3 (12.0%)	13 (16.7%)	0.649
Indication	Disabling claudication	3 (22.2%)	27 (25.2%)	0.971
	Critical limb ischaemia (rest pain and tissue loss)	14 (77.8%)	80 (74.8%)	0.355
Bypass type	Femoropopliteal	10 (55.5%)	64 (59.8%)	
	Femorodistal (peroneal - 5, ATA - 2, PTA - 1)	8 (44.4%)	38 (35.5%)	0.485
Urgency of case	Elective	12	60	0.196
	Urgent	5	45	
	Emergent	1	22	
Conduit type	Autogenous	17 (94.4%)	98 (91.6%)	0.499
	Prosthetic	1 (5.5%)	11 (10.3%)	
Immediate outcome	Amputation (BKA/AKA)	8 (44.4%)		
	Redo surgery (thrombectomy/revised bypass)	6 (33.3%)		
	Conservative	4 (22.2%)		

Nearly half (44.4%) of the patients who had graft failure were operated on as urgent or emergency cases. The majority of patients who had early bypass failure were taken back to the theatre either for thrombectomy/revised bypass (33%) or amputation (44%), while the rest were managed conservatively with their leg deemed viable based on improved flow from common femoral endarterectomy (CFEA) and profundaplasty carried out at the time of the bypass.

## Discussion

The mode of anaesthesia does not appear to influence graft failure or other complications following open lower limb revascularisation surgery between the two patient groups in our study. This is despite the fact that most of our patients who had RA had CSE anaesthesia - the first study on this topic with a marked preference for CSE. RA appears to be a popular choice in our centre with almost 50% of patients in the cohort compared to a recent study where GA was the most commonly used type of anaesthetic in up to 96% of patients [19].

This study did not show a remarkable difference in the comorbidity profile of patients chosen for either RA or GA, as the frequency of hypertension, COPD, mean body mass index (BMI), ischaemic heart disease, hypercholesterolemia, and end-stage renal dysfunction (ESRD) were similar in both groups. The grading of morbidity including respiratory, wound and cardiac complications was done in this study in view of the few numbers using the classification method by Dindo et al. with significant morbidity being grade II-IV [18]. The incidence of significant morbidity following bypass in this study in about one in four patients (28%), though high, remains comparable to similar studies, with the overall morbidity found to be 37% in a recent review [20,21]. The spectrum of complications reported included wound infection (7.8%), graft infection (2.4%), postoperative bleeding (7.4%), graft occlusions (12.0%), lymphoedema (2.9%), and surgical site seroma (2.0%). These morbidities are significant, and efforts need to be made to reduce them further as they are a major cause of readmissions and increasing cost of care following bypass [22]. The incidence of major systemic complications was quite low in this study with MI, multiple organ dysfunction, or severe hypotension associated with unconsciousness occurring in only four (2.8%) patients compared to 5.9% of patients in a similar study [20]. The use of a similar classification system for morbidities following vascular bypass across studies will make comparison easier and further aid the development of standards of care [21].

The overall 30-day graft failure rate of 14.2% in this study cohort appears to be a little higher than the contemporary series reporting 12% early graft failure rates [21]. These may be partially due to the varying complexity of patients as improvement in technical resources and increasing patient preference for minimally invasive options have led to patients with increasingly complex lesions and at older ages undergoing infrainguinal bypass procedures [23].

The finding of no significant difference in the incidence of bypass failure between the modes of anaesthesia in this study appears to reinforce the findings of other studies [12-16]. However, other authors have reported significant advantages and better outcomes with RA [9,11]. Dodds et al. were only able to demonstrate superior early graft failure rates with RA, a difference which was not maintained at 30 days, clearly agreeing with the findings of the index study [11]. Although none of these studies reported the use of CSE as in the index study, one will agree with Pierce et al. that differences in clinical and surgical variables including surgical indications, type of conduit used, outflow target arteries, quality of haemodynamic monitoring, and perioperative anticoagulation management may be the confounding factors influencing outcome [12].

The increased incidence of critical limb ischaemia and major comorbidities especially diabetes mellitus, hypertension, and ischaemic heart disease among the patients with failed bypass indicate a more complex subset of patients. Flu et al., in a multicentre study of patient and procedure-related risk factors for adverse events (AE) after infrainguinal bypass, reported patients with AEs more often were treated for CLI, had cardiac, pulmonary, renal, carotid disease, or diabetes mellitus, were smokers, and underwent a below knee bypass [24]. Most of the patients in the index study who had failed bypass fitted into this category. However, the current study did not show a remarkable difference between failure rates for femoropopliteal and femorodistal bypass. This may be related to the predominant use of autologous saphenous veins for bypass in this study. It has been shown that when using high-quality veins, the level of the distal anastomosis (popliteal, tibial or pedal) does not affect bypass outcomes [25].

Our overall mortality of 1.6% favourably compares with the 2018 UK national recent risk-adjusted mortality rates at 2.6% for infrainguinal bypass [26]. The two mortalities in our study had cardiac complications leading to multiorgan failure, a reported major cause of death in a recent review [5]. Our findings are in line with previous studies, which have not demonstrated any differences in mortality based on the mode of anaesthesia [16,17,19]. Improvement of perioperative vascular anaesthesia care and case selection in recent years may be contributory to lower mortalities following bypass overall.

The main limitations of this study include the retrospective nature and lack of randomisation. Retrospective studies are prone to incomplete data entry, as demonstrated by the lack of records in 14 of our original cases. One is limited to working with measurements made, often for another purpose; for instance clinical care, rather than for research purposes. This might decrease, in some instances, the accuracy of specific data collected [27].

## Conclusions

The mode of anaesthesia (GA or RA) did not influence survival, respiratory, cardiac, graft failure, wound, or length of stay in this study. RA (CSE) would be an important consideration in patients who require lower limb bypass surgery but do not tolerate GA.

The presence of cardiac comorbidity was strongly associated with bypass failure and this points to a need for a standard care protocol to optimise perioperative care for better outcomes. Having established the safety of RA, especially CSE for these patients, there is an indication for multicentre prospective studies in the current era of new PAD management to investigate possible factors influencing bypass success and which subset of patients will most benefit from RA as compared to GA.
